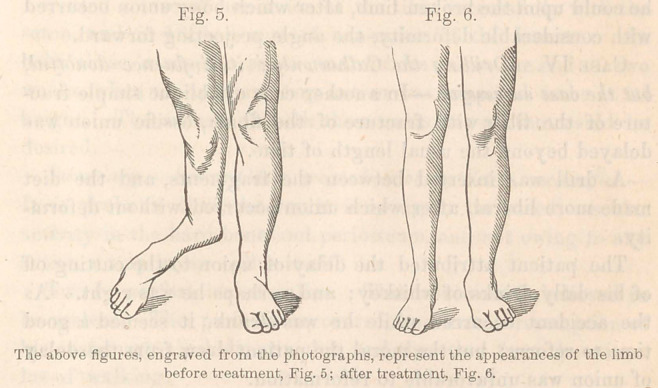# Delayed Union of Fractures, with Cases and Illustrations; the Successful Employment of Malgaigne’s Spike in Connection with Drilling, in a Case Which Had Previously Resisted Drilling Employed by Itself

**Published:** 1863-12

**Authors:** David Prince

**Affiliations:** Jacksonville, Ill.


### CHICAGO MEDICAL EXAMINER.

N. S. DAVIS, M.D., Editor.

VOL. IV. DECEMBER, 1863. NO. 12.

### Original Contributions.

ARTICLE XXXIII.

### DELAYED UNION OF FRACTURES, WITH CASES AND ILLUSTRATIONS;

### THE SUCCESSEUL EMPLOYMENT OF MALGAIGNE'S SPIKE IN CONNECTION WITH DRILLING IN A CASE WHICH HAD PREVIOUSLY RESISTED DRILLING EMPLOYED BY ITSELF.

By DAVID PRINCE, M.D., of Jacksonville, Ill.

Read to the Illinois State Medical Society.

While fractures sometimes unite under the most adverse circumstances, at other times union is delayed or does not take place where the appearances are at first most favorable. This difference of results, independent of external circumstances, can only be accounted for by the assumption of constitutional differences of aptitude to bony formation. While ossification will sometimes extend through an inch or two of plasma, reaching from one fragment to another, the separation to the extent of one-fourth of an inch will at other times prevent the bony union of the fragments. While privation and starvation sometimes fail to retard union, there is in some constitutions a necessity for a liberal diet to afford the necessary stimulus to bony deposit. The antiphlogistic remedies for high inflammation, if continued unnecessarily long, may sometimes prevent union, while in other instances no practical amount of local or general reduction will interfere with bony formation. While, therefore, it is never safe to omit any of the conditions of success in the treatment of fractures, the greatest number of unfavorable circumstances may be insufficient to cause failure if the ossific tendency be strong.

It is suspected that a fuller investigation will show that separation of the fragments to a distance of one-fourth of an inch or more from each other, and insufficiently nutritious diet at the period of from three to five weeks from the date of injury, are the most frequent causes of delay or absence of union. If this shall be affirmed by experience, it follows that the two most important points for the surgeon to attend to are, the apposition of the broken surfaces of the fragments, and the proper nourishment of the patient during the ordinary period of ossification. It must not be forgotten, however, that the extreme of fulness in diet may beget conditions of the system more dangerous and unwelcome than protracted non-union.

The delay having occurred, and the fragments remaining beyond the usual period, connected by soft callus of a greater or less degree of firmness, the treatment will at once suggest itself to secure local stimulation by frictions upon the skin, movement of the broken surfaces upon each other, a resort to more liberal diet, securing a better general health by exercise or exposure in the open air, and pressure upon the parts with reference to the approximation of the fragments when this is practicable, and when the delay may be suspected to depend upon the motion of the fragments upon each other, the diminution or arrest of this motion.

All these failing, some means of inducing more active capillary circulation with congestion or inflammation must be resorted to.

1. In the list of means to this end, is the passing of a seton through the callus between the fragments. This may be supposed to excite inflammation in all the parts immediately surrounding the seton, including the neighboring periosteum. As an important point of treatment is to get the action of ossification started somewhere, in order to favor the propagation of this action through the fibrinous material constituting the callus, the treatment is based upon intelligible physiological principles.

From the known tendency of long-continued inflammation in and near the periosteum, to induce bony deposit, it may be that Dr. Physick was right in retaining the seton a long time, with the result of a protracted congestion in the neighboring bone and periosteum. It may be in practice better to try the seton first for the short period, and, if that fails, to try it for the long period where this method of treatment is pursued.

2. The injection of some stimulating agent like iodine into or around the callus is founded on correct principles, but must be so extremely uncertain that in the possession of surer means it is not worth any further trials.

3. Electricity or galvanism passed through acupuncture needles introduced into the substance between the fragments, or in close proximity to them, can only be expected to succeed by exciting hyperæmia or inflammation.

4. Opening the parts, and scraping or sawing off the ends of the fragments, converts the case into one resembling compound fracture; but in very old cases, in which the false joint resembles a capsular ligament with its inclosed synovial membrane and cavity, this severe proceeding may be necessary. In any case in which the duration of the false joint is not measured by years, it is not easy to conceive this process to be necessary.

Dieffenbach's method of drilling and introducing ivory plugs, leaving them there to excite suppuration, can hardly be conceived better than the seton carried through the soft callus between the bones, while the risk of necrosis must be a strong objection to the proceeding.

6. Brainard's method of drilling through the solid bones and their intermediate soft callus, so practicing the operation as to permit the skin to slide over the opening when the drill has been withdrawn, has two theoretic recommendations. First, a very great disturbance of the particular portions of the bone drilled is effected, giving rise to the production of new plastic material for the formation of callus in the track of the drill, without the occurrence of suppurative inflammation. Suppuration here, as in the healing of other tissues, must be supposed to retard the union, though the active capillary circulation in the vicinity of its seat may result in subsequent ossification.

The case may be thus stated: If the bony deposit can be induced by congestion or non-suppurative inflammation, it is more speedy than that brought about by suppurative inflammation. Yet there may be cases in which a long-continued inflammation with suppuration will induce the formation of bone after the failure of a shorter course of inflammation without suppuration.

In the cases in which there can be success by congestion or non-suppurative inflammation, suppuration is an evil retarding the result. In the other cases it is a necessary attendant upon the prolonged inflammation.

Second. When the operation results in the effusion of plastic lymph without suppuration, there are new centres of ossification in the chips of bone cut off by the drill. These are left in the track of the drill; some of them in the soft callus between the ends of the fragments.

That these minute fragments of bone become parts of the living tissue which organizes around them is certain; for, if they did not, they would, by the offensive emanations of dead bone, excite suppuration and work their way to the exterior. The importance of these little fragments cut off by the drill, as centres of ossification, may have received too little attention. As in crystallization, the introduction of a single minute crystal may be sufficient to start a process which is backward to commence without catalytic aid, so the process of ossification, when slow to begin, may be set in operation by a fragment of bone or periosteum imbedded in the plastic material. To obtain this advantage of the bony fragments it is, of course, necessary that suppuration in the track of the drill should be avoided.

7. Applying metallic wires around the fragments to approximate them and prevent lateral motion, answers an obvious indication. To apply a wire around the fragments, it is, however, necessary to convert the fracture into the condition of a compound fracture, and afterwards, when union has taken place, the wire is to be left in, or removed at the expense of much disturbance of the parts. If a silver, gold, or platinum wire becomes covered with organized lymph or granulations it can do no harm, and may be allowed permanently to remain.

8. Perhaps a bone might be drilled through both fragments and held in apposition by a rivet of one of these metals. The presence of the rivet after the completion of the healing process would do no harm, and if a permanent discharge should be the result the metal could be readily removed.

9. Metallic points arranged for pressure on one or more of the fragments for the purpose of approximating them.

This expedient, where the nature of the parts makes it practicable, supplies an important indication. It accomplishes all that can be secured by the application of wires with more certainty, without extensively disturbing the soft parts, and the apparatus is easily tightened or loosened, increasing or diminishing the pressure, and is easily removed altogether.

Whether the separation of the fragments has been occasioned by the action of muscle or by the interposition of muscle or other material, the pressure will be constant, tending continually to approximate them.

Malgaigne's single spike for oblique fracture of the lower portion of the tibia is intended to prevent what it may afterwards be employed to remove, *i. e.*, a too wide separation of the fragments. In this apparatus the counter pressure is by means of a strap passing round the leg, including a splint, which distributes the pressure upon the back of the leg. In other cases the counter pressure must be by means of opposing points acting upon the opposed fragments, in order to bring them into close contact. Skill in making and adjusting the apparatus will be chiefly exercised in making it occupy sufficiently small space not to be in the way of placing the limb alternately in various positions while the process of union is going on.

Wherever the application of pressure by metallic points penetrating the soft parts and pressing the bony fragments together becomes necessary, it would have been important to apply them in the first place to bring the fragments into close contact and favor union by what is termed by PAGET *immediate union*, or by *primary adhesion*.

This is a new treatment, and the reason why it has not been adopted before this time is probably the repulsive appearance of the treatment to patients and friends. It is found by experience, however, that very little pain is occasioned by wearing for weeks a steel point, applied with considerable force, to the fragment to be held.

The treatment does not convert the fracture into the condition of a compound fracture, for the point can be applied at a sufficient distance from the place of fracture to avoid this complication. When, however, points have to be applied to opposite sides of the limb to act upon different fragments at the same time, they must be nearly or quite opposite each other; but as it is only in oblique fractures that the treatment is admissible, it will only in very rare cases be necessary to penetrate the interior wound in the soft parts.

In cases of compound fracture, the points can be introduced into the wound or through the uninjured soft parts, as may be most convenient. This, as a first treatment of fracture, may be found to be less painful than apparently more comfortable modes of dressing, obviating the movement of one fragment upon the other by the closeness with which the surfaces are brought together. Some periosteal inflammation must be excited, which, if it extends to the fractured lines, can only the more certainly result in bony formation, whether as a primary treatment or as a method of curing non-union. A slight exfoliation of bone may occur at the spot where the metallic point is made to press; but this is a trifling consideration in comparison with an increased efficacy in the treatment.

Fig. 1.

Modification of Malgaigne's spike, employed for delayed union in oblique fractures.

A single point may be applied by means of the metallic yoke and strap, as employed by Malgaigne, and where two or more points are to be applied on opposite sides of the limb, an apparatus may be constructed resembling the clamp used by ladies to fasten to a table any fabric for greater convenience in sewing upon it, or like some forms of tourniquet made to apply opposing pads by means of a steel yoke approximated by some screw arrangement. The pads would for this purpose be replaced by points. The apparatus should be so arranged as to be capable of compressing the fragments as closely as may be necessary to keep them in apposition, and to hold them without any yielding whatever. There should be no elasticity in the retaining apparatus. (Fig. 1.)

If the pressure of the fragments upon each other is found to be painful to the patient, the screw may be loosened a very little, as a very small relaxation of pressure will be capable of affording relief.

CASE I. *Non-union of Tibia unsuccessfully treated by Drilling; afterwards successfully treated by Drilling followed by Compression of the Fragments by means of Malgaigne's Spike.*—Lt. Samuel L. Hamilton, Co. F, 19th Regt., Illinois Volunteers, on the 15th of May, 1862, had both fibula and tibia of the right leg broken, a short distance above the ankle, by being thrown from a wagon, lighting upon his feet. He was treated in the army hospital, and the patient says his surgeons had considerable difficulty in keeping the bones in proper position.

After a few weeks, a starch bandage was applied, and the patient went upon crutches. The fibula united by bony material, but the tibia remained ununited. Some deformity existed from the action of the muscles, sliding the lower fragment upon the upper and bending the fibula, bringing the outside of the foot to the ground.

*Operation under Ether by Drilling, after Brainard's Method,* Nov. 5, 1863, five months and twenty days from the date of the injury:—The fragments of the tibia were forcibly moved upon each other, and two holes were drilled through both fragments and the intermediate soft callus. The callus seemed, from the jumping of the drill, to be a-quarter of an inch in thickness.

A side splint was applied, extending from the upper portion of the tibia over the malleolus, around which the limb was firmly bandaged. The fibula thus received the whole force of the bandage on one side, while upon the other side, the force of the bandage was received upon the malleolus, and the upper portion of the tibia by the intermedium of the splint. In two weeks the constant pressure had straightened the fibula so that there was no deformity. There was no perceptible motion between the fragments, and the splint was directed to be worn some time longer with the expectation of success.

This operation proved a failure, and the movement of the fragments upon each other became obvious enough.

*Second operation: Drilling and the Application of Malgaigne's Spike*, March 11, 1863, ten months from the injury, and four months from the previous operation:—A very obvious deformity had been reproduced. The muscles acting upon the fibula as a fulcrum, had bent it so as to bring the outer side of the foot to the ground, while the inner side was slightly lifted from it. The patient having been brought under the influence of ether, the fibula was forcibly straightened by interstitial breaking, or by bending with breaking of portions of the substance; after which a-quarter-inch drill was introduced between the fragments passing from below upward and backward, and freely rotated in the space between the two fragments, breaking up the soft intervening callus. The fragments were thus shown to be one-quarter of an inch asunder. A small probe was introduced and left as the drill was withdrawn. Three holes were then drilled through the anterior fragment and intermediate callus and into the posterior or lower fragment.

The limb was then put upon a posterior splint which was a double inclined plane, and the steel-point of Malgaigne's spike placed about an inch above the lower end of the upper fragment, through an incision made in the skin by a bistoury, the strap adjusted beneath the splint and the screw turned down until the probe left between the fragments was very firmly grasped by the approximation of the fragments. A light side-splint was applied on each side within the yoke holding the spike. The probe was then pulled out from between the fragments.

With slight adjustments from time to time this apparatus was worn without removal twenty-eight days. The patient took opium enough during the first few days to quiet pain. He was overtaken with a chill, to which he had for several months been subject, after which he had the consequent fever, with a pulse of 120. He took quinia for this, and lager beer. As soon as he was free from his ague he discontinued medicine. Considerable swelling and suppuration occurred around the spike, which was not attended with much pain. The apparatus looked worse than it felt.

*April 8th.* The twenty-eighth day removed the dressing, and applied a tin side-splint.

17*th.* Applied a starch bandage, which was split on the 19th, and directed to be worn two weeks longer.

There is a node on the inner side of the tibia, exactly opposite the point occupied by the spike, as if periosteal inflammation had extended around the limb from the point of irritation by the spike. The minute exfoliations afterwards came out in the vicinity of the point pressed upon by the spike. Consolidation followed this treatment, without impairing confidence to the patient, who cautiously ventured to walk upon the limb. The patient left to rejoin the army the first of July.

(Fig. 2.)

Appearance of leg of Lieut. Hamilton, March 10, 1863.

(Fig. 3.)

Appearance of leg of Lieut. Hamilton, June 27, 1863.

(Engraved from Photographs.)

CASE II. *Ununited Fracture of Tibia and Fibula of three years' duration, with much Angular Deformity from Contraction of Muscles. Reduction of Deformity by Extension and Lateral Pressure—Drilling the Bones according to Brainard's Method, resulting in Bony Union without deformity or Lameness.*—Augustus Simpkins, of Pike County, Illinois, aged about thirty-five years, had a simple transverse fracture of the middle portion of the tibia and fibula of the right leg, by the fall of a tree.

There is said to have been much swelling and inflammation, and the skin was cut to let out the effused fluids. Cold applications were kept upon the leg, and the patient restricted to a low diet. No union by bone followed, and the angular deformity—the foot being carried out, making the leg look like a limb with a knock-knee—resulted gradually from muscular contraction. When the patient stands erect the toes only come to the ground, the lower portion of the leg being at an angle of 45° with the other leg.

Fig. 4.

A. Screw with its concave pad applied to the projecting angle of the leg. B. Hook for retaining the screw, making counter-pressure upon the splints. C. Long splint, which is the medium of extension. D. Back splint attached to the long splint, for aiding in securing the counter-lateral pressure.

*June* 12, 1861. The non-union has been of three years' duration. Applied the most powerful extension practicable by the lever arrangement of Jarvis' adjuster attached to the distal end of a long splint, the counter-extension being upon the ischium and groin, while lateral pressure was applied by a sort of tourniquet working with a strong screw.

Forcible working of the ends of the bones upon each other was practised by taking hold of the limb with the hands, and the tendo-Achillis was divided. With all this the limb was not restored to its straight position, and the apparatus breaking under the great strain applied, the process was stopped. The limb was dressed so as to retain as far as possible what had been gained.

After five days, not much inflammatory excitement had appeared, and the limb was subjected to another process. The bones were drilled from one fragment into the other in six places, taking different directions, all traversing the soft callus between the ends of the bones. The extension and lateral pressure were applied as in the first instance, only with stronger apparatus. The extension was from the ankle, by means of a roller applied around it to hold the loops. The limb was not only straightened by this operation, but the muscular resistance was so completely overcome that I bent the limb in the opposite direction without difficulty. The thigh, leg, and foot were then placed in a side-splint, made of tin, and kept in it until the consolidation was complete, except when taken out for washing and friction to the skin.

In three weeks from the first operation he went home, a distance of 40 miles, riding about half the way in a buggy. The splint was worn about ten weeks. Perhaps it might have been laid aside sooner, but the patient, after three years' experience, was afraid to trust his limb too soon.

During the operation, a mixture of ether and chloroform was inhaled, and, to quiet the subsequent pain, morphia was freely administered. No other antiphlogistic treatment was resorted to than cathartics.

Fig. 5. Fig. 6.

The above figures, engraved from the photographs, represent the appearances of the limb before treatment, Fig. 5; after treatment, Fig. 6.

The result in this case should lead us never to dispair of success, until after trials of means of cure. As the fracture of the tibia was transverse the interposed substance was subjected to great pressure by the contraction of the muscles, and there was no want of apposition to account for the non-union. It is suspected that the antiphlogistic treatment was too long continued. The fragments of the fibula became overlapped as the limb assumed the angular position, but when brought into proper relations by straightening the limb the fragments became united by bony substance.

The preceding figures (Figs. 5 and 6) represent the conditions before and after treatment.

CASE III. *Drilling the Callus only, unsuccessful; Bony union afterwards induced by walking.*—In one case of simple oblique fracture of the upper portion of the lower third of the tibia and fibula by the falling of a tree, originally treated by me with great care by extension to avoid shortening or other deformity, the ossific union was delayed beyond the usual time. The callus was broken up by the insertion of a drill between the fragments of bone, but the hard bone was not drilled. This means failed up to the time when the patient, becoming impatient, placed himself in charge of another practitioner, who removed the splint and set the patient to exercising, bearing what weight he could upon the broken limb, after which bony union occurred with considerable deformity, the angle projecting forward.

CASE IV. *Drilling the Callus only; its influence doubtful, but the case successful.*—In another case of oblique simple fracture of the tibia with fracture of the fibula, ossific union was delayed beyond the usual length of time.

A drill was inserted between the fragments, and the diet made more liberal, after which union occurred without deformity.

The patient attributed the delay of union to the cutting off of his daily drinks of whiskey; and perhaps he was right. As the accident occurred while he was drunk, it seemed a good time to reform; but the moral the patient drew from the delay of union was unfavorable to reformation.

I am led to think that the perforation of the callus by awls or drills, which do not penetrate the bony substance, is useless, and perhaps worse than useless, by breaking up its organization without influencing the bone and periosteum, whence the process of bone formation most readily proceeds.

CASE V. *Drilling the Bone.*—Thomas Mulready, an Irishman of short stature, aged about thirty, had an oblique fracture of the lower third of the tibia, beginning two and a-half inches above the joint, and extending upwards and backwards with fracture of fibula.

I first saw the case three months after the injury, when there was forward projection of the upper fragment of the tibia, with a shortening of an inch and three-quarters. The fibula had united.

Four holes were drilled through both fragments and the intermediate soft callus. Side-splints made of cloth, saturated with an alcoholic solution of shellac, were applied and worn twenty-two days from the date of the perforation, when the fragments were found to have become consolidated. During a part of this time the patient was pretty well stimulated with whiskey and quinine.

The recovery was complete and permanent.

CASES VI. & VII. *Seton successful in two Cases.*—In 1848, I treated a case of non-union of the tibia successfully with the seton, and in 1851, a case of non-union of the humerus. In both these cases the seton was withdrawn at the end of two weeks, when the inflammatory action was supposed to be at its height. The success in both these cases was all that could be desired.

*Summary. Seven Cases treated—two by Seton successful.*—It is probable that the result is owing to increased vascular activity in the hard bone and periosteum, and not owing to any action set up in the callus itself.

Two by perforation of callus. This treatment is believed to be useless. The patients recovered, one from a resumption of his customary alcoholic stimulus, and the other from the stimulus of walking.

Three by drilling through the hard bone of both fragments. Two of these cases were successful on first trial; the other was unsuccessful at first, but afterwards successful when combined with compression by means of the metallic point impinging upon the projecting fragment.

Of the seven cases, all ultimately recovered; the two in which the callus was simply perforated would probably have done as well without the perforation.

Six of the cases were of the leg, and in all of them both bones were originally broken.

In four of the six cases, the fibula united while the tibia remained ununited. In two cases, the fibula remained ununited until the tibia finally united, but united at length without any treatment applied directly to the fibula itself. From this it appears that the fibula is more prone to unite than the tibia. Perhaps this is because the fracture is more likely to be transverse, on which account it is less subject to displacement, and because the tendinous and muscular investments hold the two fragments of the fibula together instead of tending to separate them, as is the case in oblique fractures of the middle and lower portions of the tibia.

One case of the middle portion of the humerus. The seven cases all ultimately successful.

—

Note. Oct. 20, 1863.—Since this article was written, I have treated two cases of delayed or tardy union. Both of these cases were oblique fracture of the middle third of the tibia, with fracture of the fibula, the small bone uniting in the usual period.

In one of these cases the fracture was compound, and the spine of the lower end of the upper fragment projecting anteriorly, and rising one-third of an inch from its corresponding surface on the upper end of the lower fragment, and overlapping or shortning about half-an-inch; there was no bony union at the end of nine weeks. The spike was applied upon the exposed spine of the upper fragment, pressing it very firmly upon the lower fragment, after which bony union speedily occurred. The pressure by the metallic point removed deformity at the same time that it secured union.

In the other case there was no bony union at the end of twelve weeks from the date of the injury.

*June* 10. A starch bandage was applied, and the patient was set to walking with crutches, hoping to obtain union by the stimulus of exercise.

*June* 24. Fourteen weeks from the date of injury. No diminution of movement of the fragments upon each other, having been secured by this means, the bone was drilled.

In this operation a small drill was first made to penetrate between the fragments, from below upward and backward, through the whole oblique diameter of the bone, showing that the fracture, which had been supposed to be transverse, was oblique. This drill was left in position to serve as a guide for the place and direction of the holes to be made through the bony fragments. Four holes with a-quarter-inch drill were made through both fragments, traversing the soft callus between them.

A starch bandage was again applied. Two holes suppurated and two did not.

The patient wrote, date Oct. 4th: "I commenced to walk on the leg about the 20th of August, with a cane." (This is nearly ten months from the time of the drilling.) "I can now go without a cane, but I carry one. The leg is very hot at times and very sensitive; and if I step on a stick in the hollow of my foot it hurts, but in walking in smooth ground I experience no difficulty. My ankle is still weak, and that hurts more than the leg. That leg is a little the shortest."

It will be noticed in this case, that the stimulus of exercise with the limb in a dependent position from the 12th to the 14th week from the date of the injury, failed to diminish the mobility of the fragments upon each other. The ordinary period for bony deposit having passed by, it was difficult to reinstate the process of bone-formation.

## Figures and Tables

**Fig. 1. f1:**
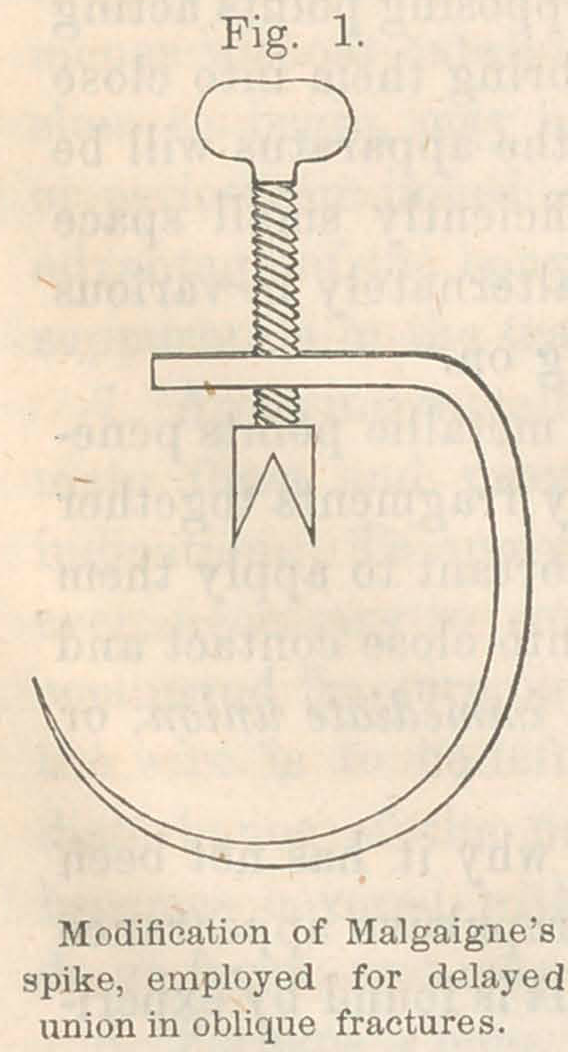


**Fig. 2. Fig. 3. f2:**
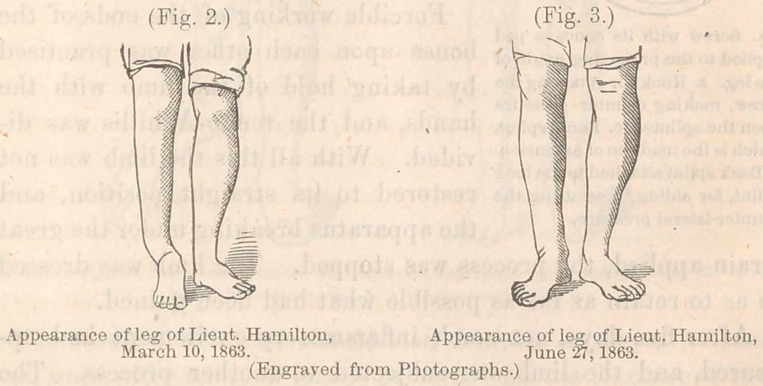


**Fig. 4. f3:**
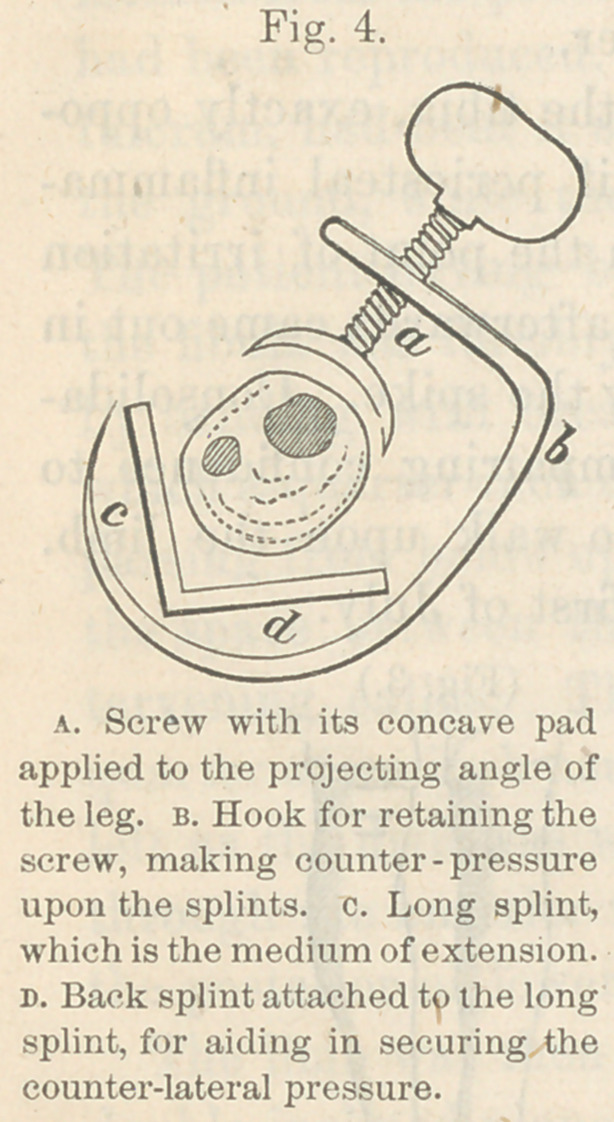


**Fig. 5. Fig. 6. f4:**